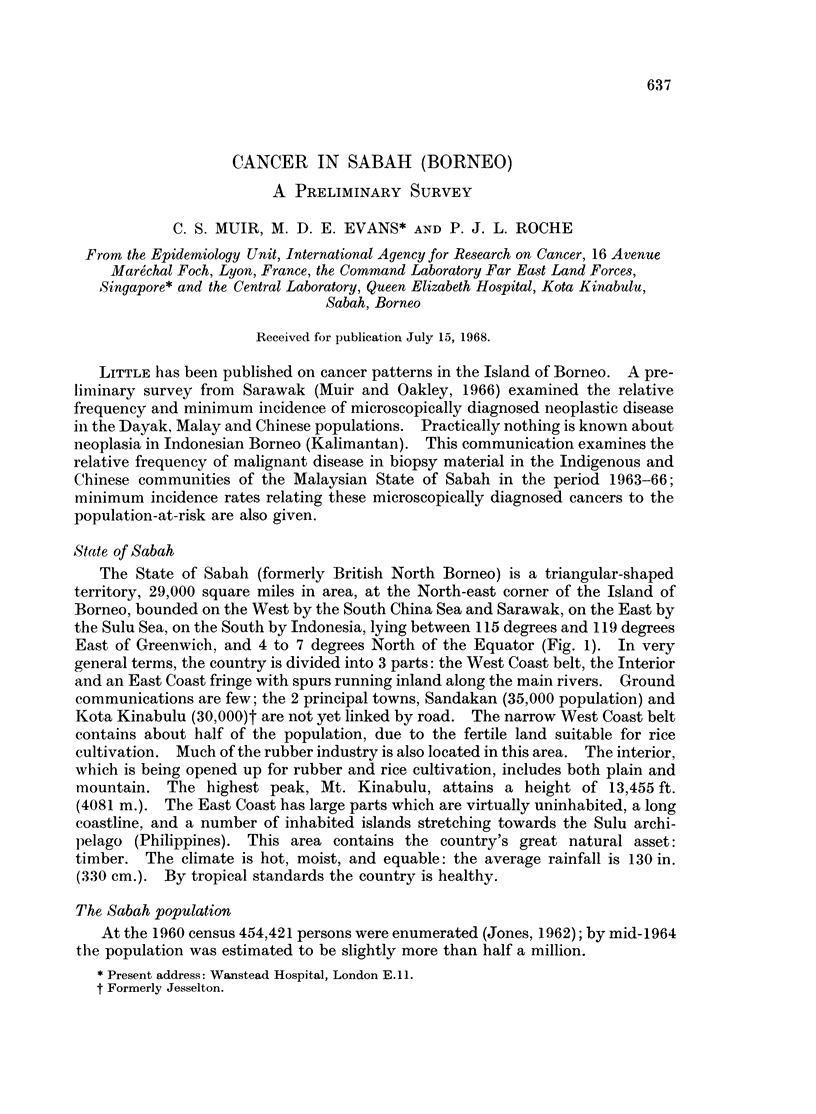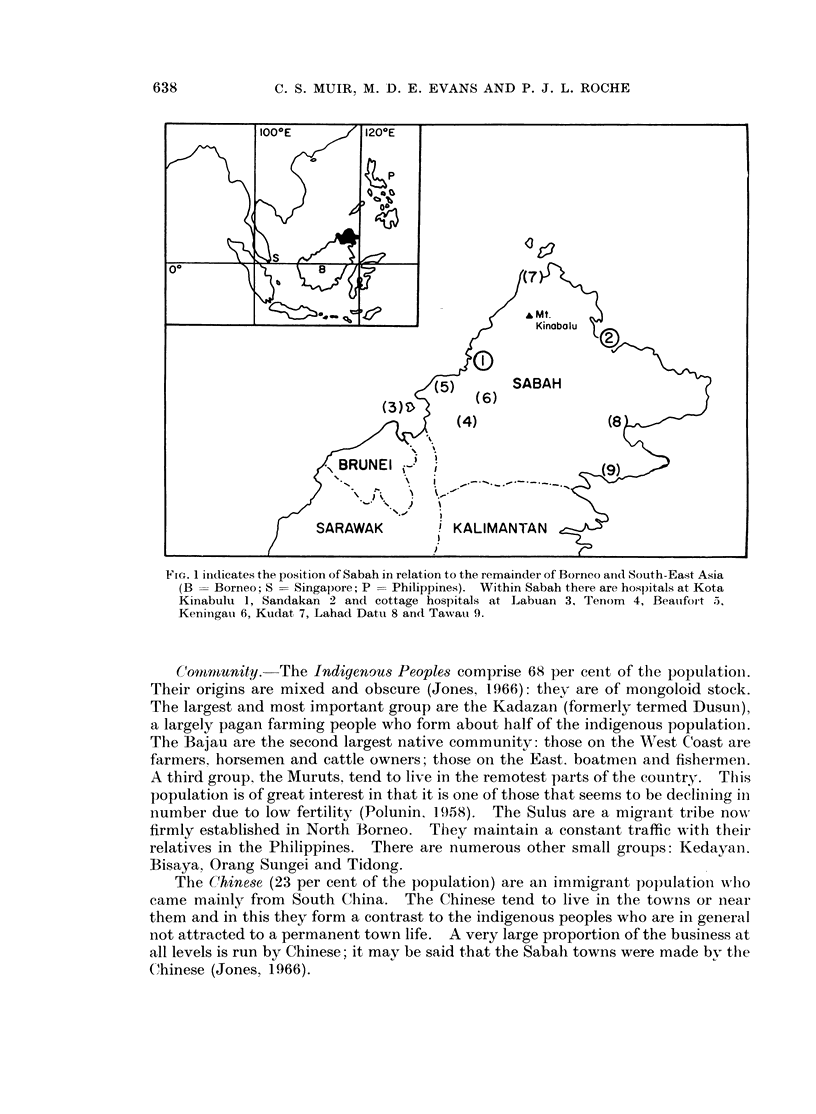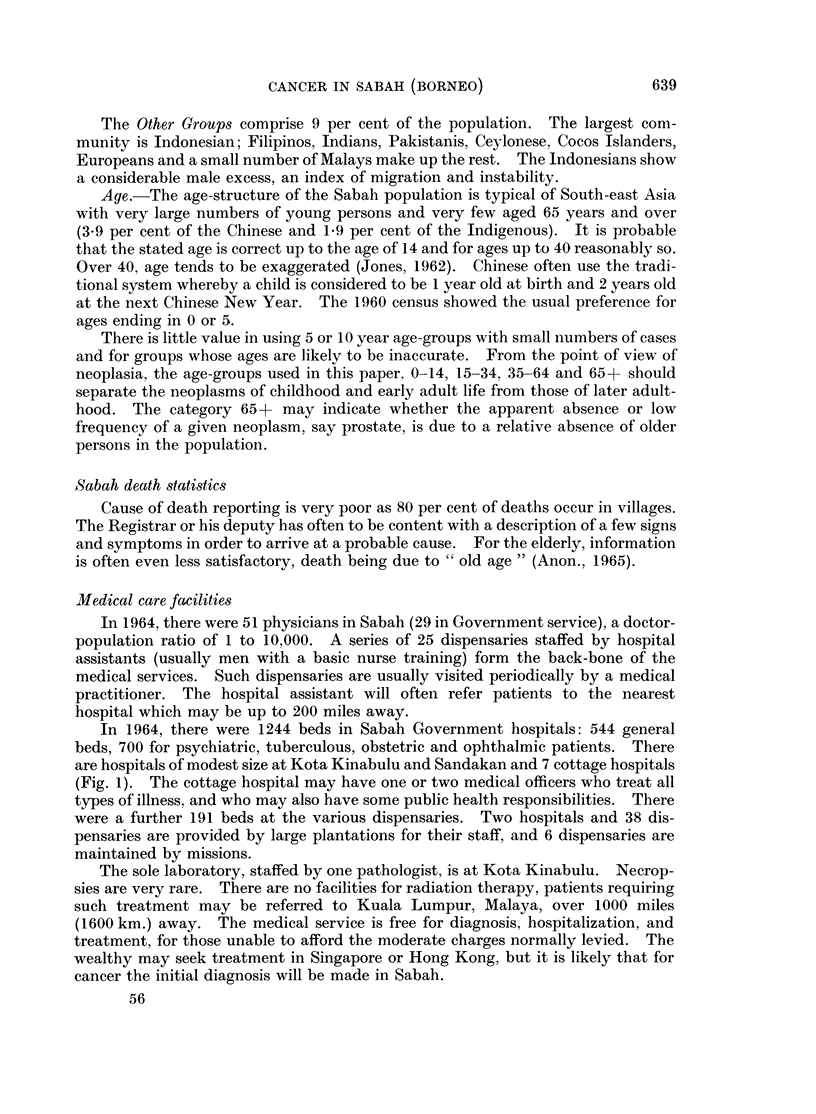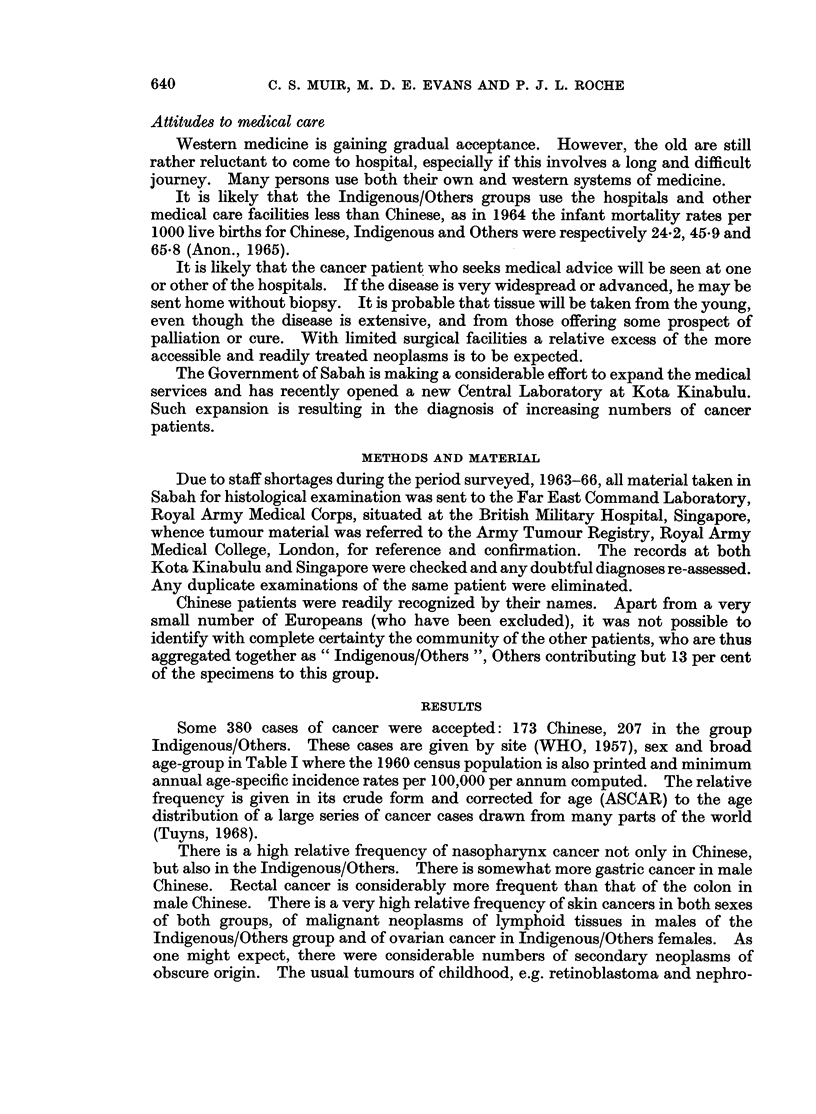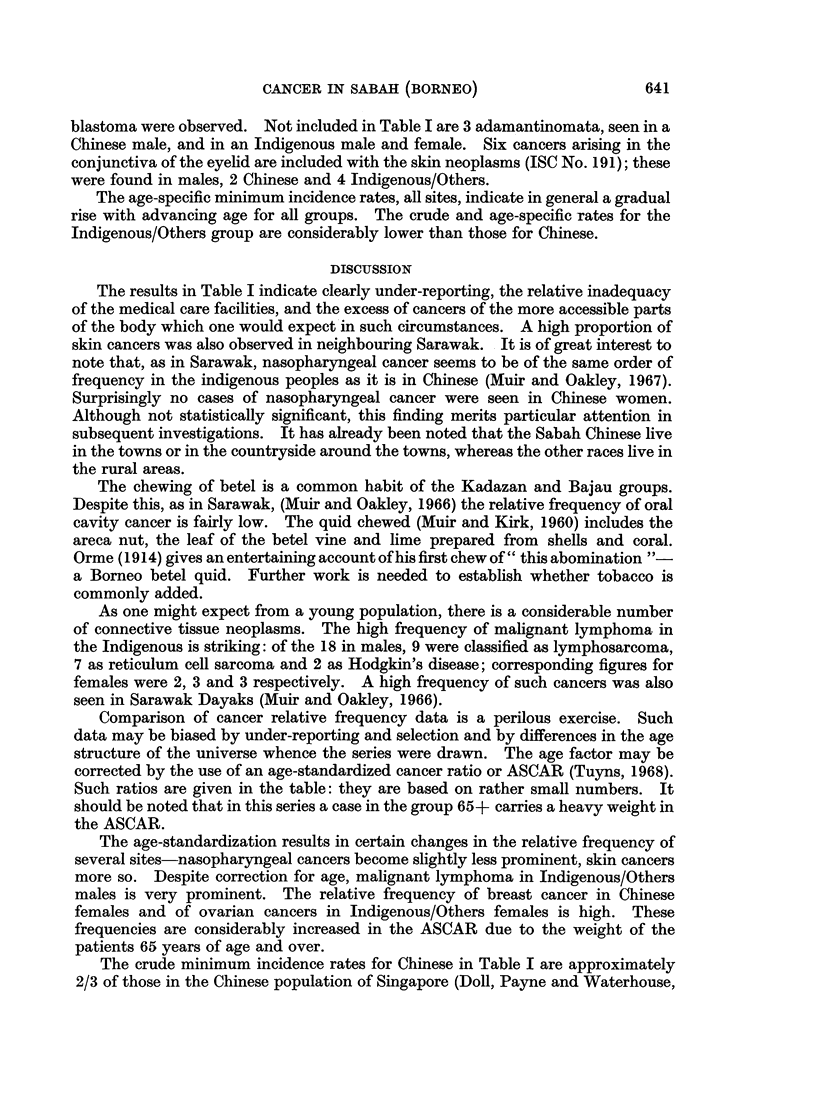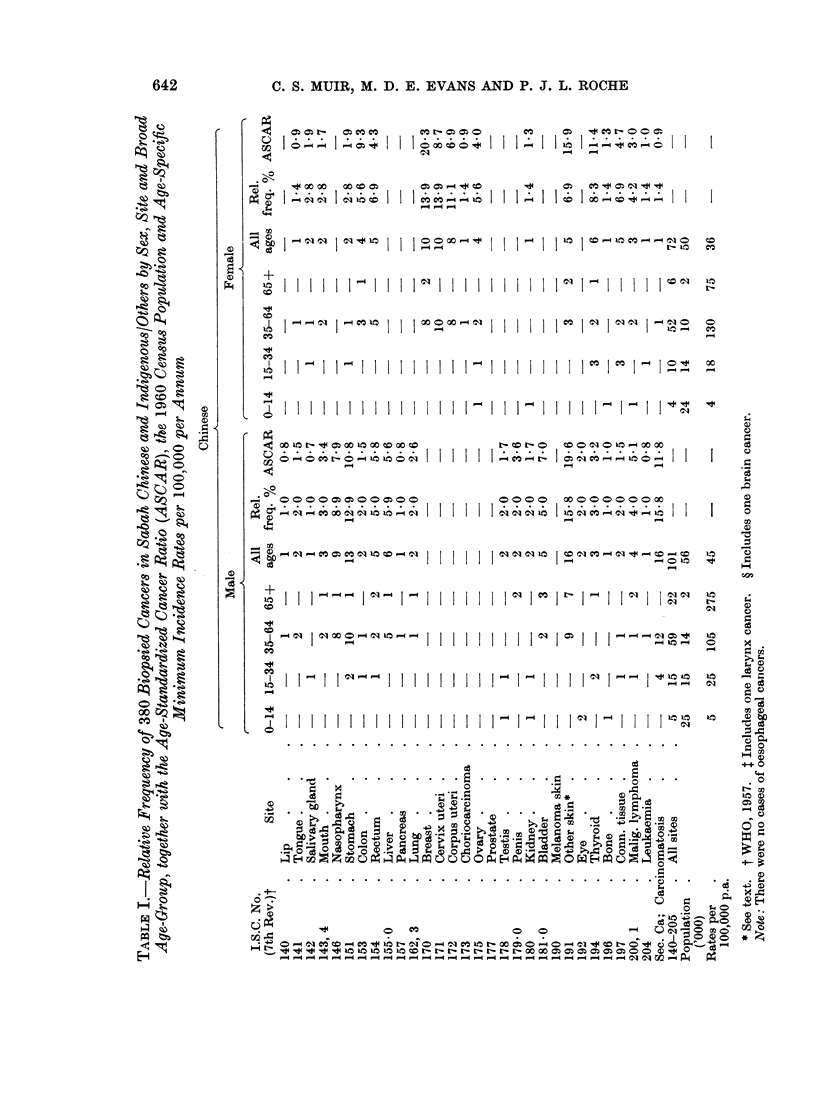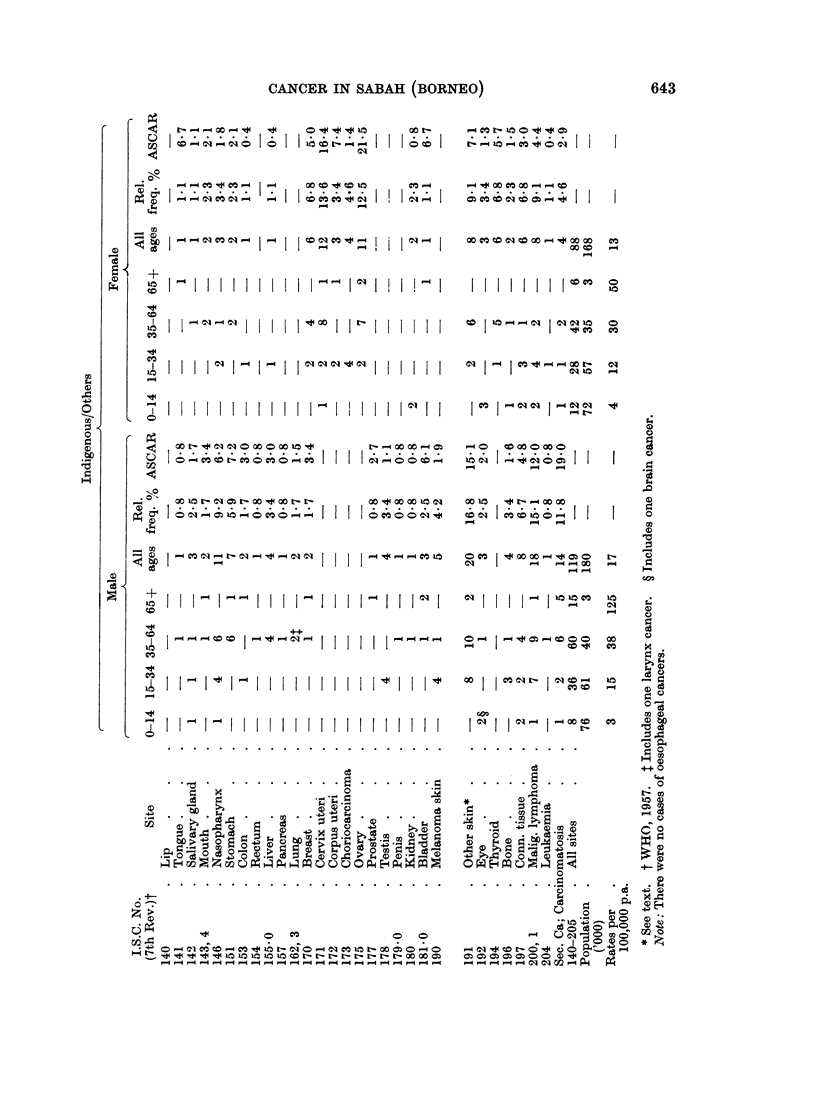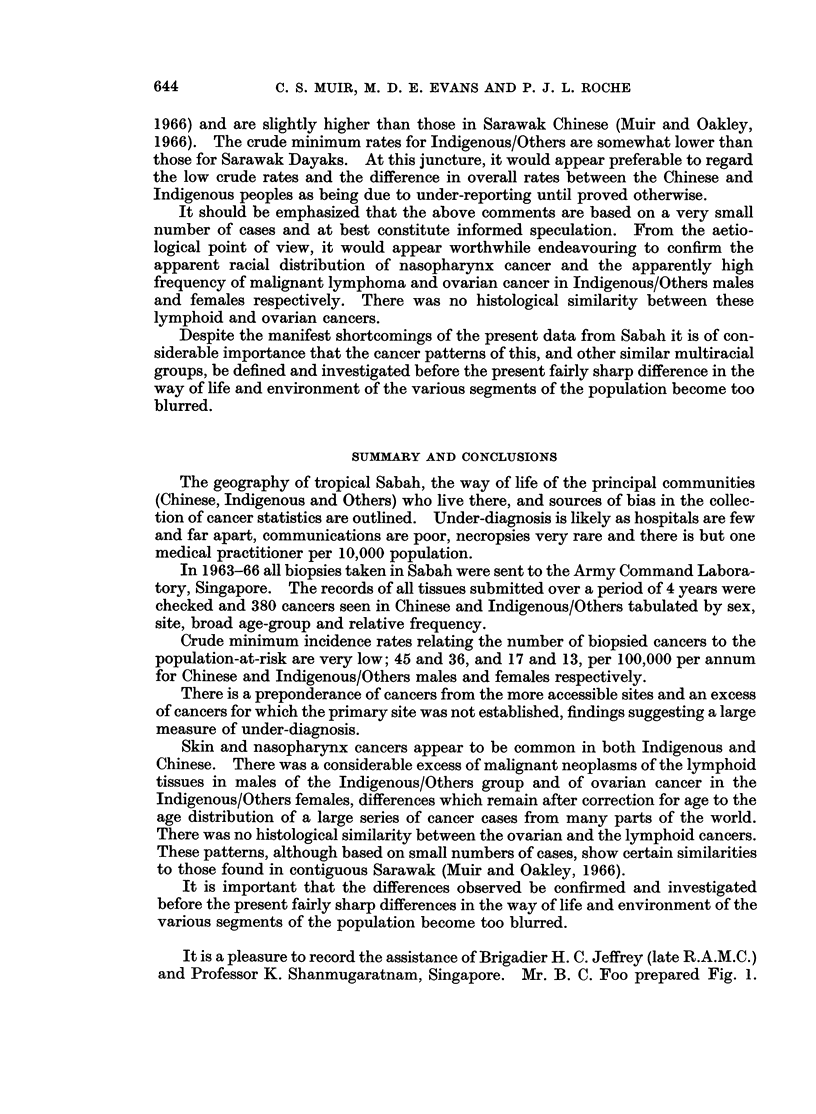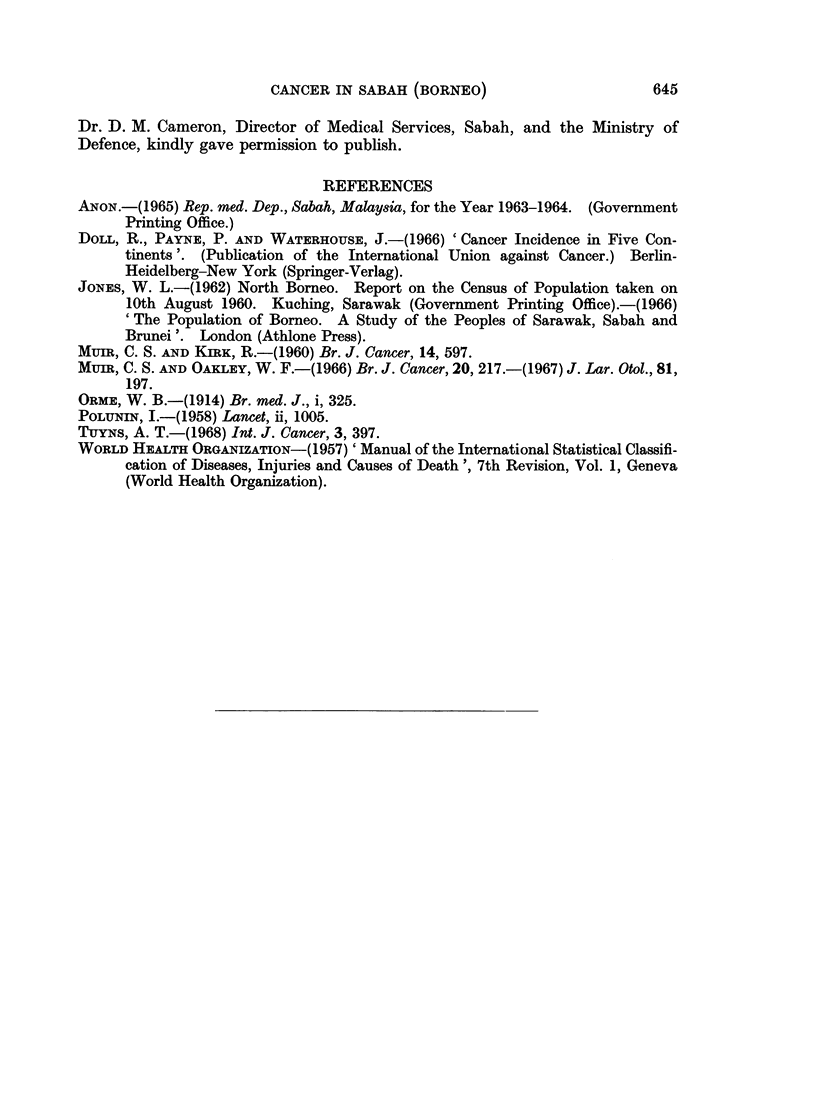# Cancer in Sabah (Borneo) a prelimanry survey.

**DOI:** 10.1038/bjc.1968.75

**Published:** 1968-12

**Authors:** C. S. Muir, M. D. Evans, P. J. Roche


### CANCER IN SABAH (BORNEO)

### A Preliminary Survey

C. S. MUIR, M. D. E. EVANS* and P. J. L. ROCHE

*From the Epidemiology Unit, International Agency for Research on Cancer, 16 Avenue Maréchal Foch, Lyon, France, the Command Laboratory Far East Land Forces, Singapore* and the Central Laboratory, Queen Elizabeth Hospital, Kota Kinabulu, Sabah, Borneo*

Received for publication July 15, 1968.

Little has been published on cancer patterns in the Island of Borneo. A preliminary survey from Sarawak (Muir and Oakley, 1966) examined the relative frequency and minimum incidence of microscopically diagnosed neoplastic disease in the Dayak, Malay and Chinese populations. Practically nothing is known about neoplasia in Indonesian Borneo (Kalimantan). This communication examines the relative frequency of malignant disease in biopsy material in the Indigenous and Chinese communities of the Malaysian State of Sabah in the period 1963–66; minimum incidence rates relating these microscopically diagnosed cancers to the population-at-risk are also given.

### *State of Sabah*

The State of Sabah (formerly British North Borneo) is a triangular-shaped territory, 29,000 square miles in area, at the North-east corner of the Island of Borneo, bounded on the West by the South China Sea and Sarawak, on the East by the Sulu Sea, on the South by Indonesia, lying between 115 degrees and 119 degrees East of Greenwich, and 4 to 7 degrees North of the Equator (Fig. 1). In very general terms, the country is divided into 3 parts: the West Coast belt, the Interior and an East Coast fringe with spurs running inland along the main rivers. Ground communications are few; the 2 principal towns, Sandakan (35,000 population) and Kota Kinabulu (30,000)† are not yet linked by road. The narrow West Coast belt contains about half of the population, due to the fertile land suitable for rice cultivation. Much of the rubber industry is also located in this area. The interior, which is being opened up for rubber and rice cultivation, includes both plain and mountain. The highest peak, Mt. Kinabulu, attains a height of 13,455 ft. (4081 m.). The East Coast has large parts which are virtually uninhabited, a long coastline, and a number of inhabited islands stretching towards the Sulu archipelago (Philippines). This area contains the country's great natural asset: timber. The climate is hot, moist, and equable: the average rainfall is 130 in. (330 cm.). By tropical standards the country is healthy.

### *The Sabah population*

At the 1960 census 454,421 persons were enumerated (Jones, 1962); by mid-1964 the population was estimated to be slightly more than half a million.

* Present address: Wanstead Hospital, London E.11.
† Formerly Jesselton.

FIG. 1 indicates the position of Sabah in relation to the remainder of Borneo and South-East Asia (B = Borneo; S = Singapore; P = Philippines). Within Sabah there are hospitals at Kota Kinabulu 1, Sandakan 2 and cottage hospitals at Labuan 3, Tenom 4, Beaufort 5, Keningau 6, Kudat 7, Lahad Datu 8 and Tawau 9.

*Community*.—The *Indigenous Peoples* comprise 68 per cent of the population. Their origins are mixed and obscure (Jones, 1966): they are of mongoloid stock. The largest and most important group are the Kadazan (formerly termed Dusun), a largely pagan farming people who form about half of the indigenous population. The Bajau are the second largest native community: those on the West Coast are farmers, horsemen and cattle owners; those on the East, boatmen and fishermen. A third group, the Muruts, tend to live in the remotest parts of the country. This population is of great interest in that it is one of those that seems to be declining in number due to low fertility (Polunin, 1958). The Sulus are a migrant tribe now firmly established in North Borneo. They maintain a constant traffic with their relatives in the Philippines. There are numerous other small groups: Kedayan, Bisaya, Orang Sungei and Tidong.

The *Chinese* (23 per cent of the population) are an immigrant population who came mainly from South China. The Chinese tend to live in the towns or near them and in this they form a contrast to the indigenous peoples who are in general not attracted to a permanent town life. A very large proportion of the business at all levels is run by Chinese; it may be said that the Sabah towns were made by the Chinese (Jones, 1966).

The *Other Groups* comprise 9 per cent of the population. The largest community is Indonesian; Filipinos, Indians, Pakistanis, Ceylonese, Cocos Islanders, Europeans and a small number of Malays make up the rest. The Indonesians show a considerable male excess, an index of migration and instability.

*Age.*—The age-structure of the Sabah population is typical of South-east Asia with very large numbers of young persons and very few aged 65 years and over (3·9 per cent of the Chinese and 1·9 per cent of the Indigenous). It is probable that the stated age is correct up to the age of 14 and for ages up to 40 reasonably so. Over 40, age tends to be exaggerated (Jones, 1962). Chinese often use the traditional system whereby a child is considered to be 1 year old at birth and 2 years old at the next Chinese New Year. The 1960 census showed the usual preference for ages ending in 0 or 5.

There is little value in using 5 or 10 year age-groups with small numbers of cases and for groups whose ages are likely to be inaccurate. From the point of view of neoplasia, the age-groups used in this paper, 0–14, 15–34, 35–64 and 65+ should separate the neoplasms of childhood and early adult life from those of later adulthood. The category 65+ may indicate whether the apparent absence or low frequency of a given neoplasm, say prostate, is due to a relative absence of older persons in the population.

*Sabah death statistics*

Cause of death reporting is very poor as 80 per cent of deaths occur in villages. The Registrar or his deputy has often to be content with a description of a few signs and symptoms in order to arrive at a probable cause. For the elderly, information is often even less satisfactory, death being due to " old age " (Anon., 1965).

*Medical care facilities*

In 1964, there were 51 physicians in Sabah (29 in Government service), a doctor-population ratio of 1 to 10,000. A series of 25 dispensaries staffed by hospital assistants (usually men with a basic nurse training) form the back-bone of the medical services. Such dispensaries are usually visited periodically by a medical practitioner. The hospital assistant will often refer patients to the nearest hospital which may be up to 200 miles away.

In 1964, there were 1244 beds in Sabah Government hospitals: 544 general beds, 700 for psychiatric, tuberculous, obstetric and ophthalmic patients. There are hospitals of modest size at Kota Kinabulu and Sandakan and 7 cottage hospitals (Fig. 1). The cottage hospital may have one or two medical officers who treat all types of illness, and who may also have some public health responsibilities. There were a further 191 beds at the various dispensaries. Two hospitals and 38 dispensaries are provided by large plantations for their staff, and 6 dispensaries are maintained by missions.

The sole laboratory, staffed by one pathologist, is at Kota Kinabulu. Necropsies are very rare. There are no facilities for radiation therapy, patients requiring such treatment may be referred to Kuala Lumpur, Malaya, over 1000 miles (1600 km.) away. The medical service is free for diagnosis, hospitalization, and treatment, for those unable to afford the moderate charges normally levied. The wealthy may seek treatment in Singapore or Hong Kong, but it is likely that for cancer the initial diagnosis will be made in Sabah.

*Attitudes to medical care*

Western medicine is gaining gradual acceptance. However, the old are still rather reluctant to come to hospital, especially if this involves a long and difficult journey. Many persons use both their own and western systems of medicine.

It is likely that the Indigenous/Others groups use the hospitals and other medical care facilities less than Chinese, as in 1964 the infant mortality rates per 1000 live births for Chinese, Indigenous and Others were respectively 24·2, 45·9 and 65·8 (Anon., 1965).

It is likely that the cancer patient who seeks medical advice will be seen at one or other of the hospitals. If the disease is very widespread or advanced, he may be sent home without biopsy. It is probable that tissue will be taken from the young, even though the disease is extensive, and from those offering some prospect of palliation or cure. With limited surgical facilities a relative excess of the more accessible and readily treated neoplasms is to be expected.

The Government of Sabah is making a considerable effort to expand the medical services and has recently opened a new Central Laboratory at Kota Kinabulu. Such expansion is resulting in the diagnosis of increasing numbers of cancer patients.

### METHODS AND MATERIAL

Due to staff shortages during the period surveyed, 1963–66, all material taken in Sabah for histological examination was sent to the Far East Command Laboratory, Royal Army Medical Corps, situated at the British Military Hospital, Singapore, whence tumour material was referred to the Army Tumour Registry, Royal Army Medical College, London, for reference and confirmation. The records at both Kota Kinabulu and Singapore were checked and any doubtful diagnoses re-assessed. Any duplicate examinations of the same patient were eliminated.

Chinese patients were readily recognized by their names. Apart from a very small number of Europeans (who have been excluded), it was not possible to identify with complete certainty the community of the other patients, who are thus aggregated together as " Indigenous/Others ", Others contributing but 13 per cent of the specimens to this group.

### RESULTS

Some 380 cases of cancer were accepted: 173 Chinese, 207 in the group Indigenous/Others. These cases are given by site (WHO, 1957), sex and broad age-group in Table I where the 1960 census population is also printed and minimum annual age-specific incidence rates per 100,000 per annum computed. The relative frequency is given in its crude form and corrected for age (ASCAR) to the age distribution of a large series of cancer cases drawn from many parts of the world (Tuyns, 1968).

There is a high relative frequency of nasopharynx cancer not only in Chinese, but also in the Indigenous/Others. There is somewhat more gastric cancer in male Chinese. Rectal cancer is considerably more frequent than that of the colon in male Chinese. There is a very high relative frequency of skin cancers in both sexes of both groups, of malignant neoplasms of lymphoid tissues in males of the Indigenous/Others group and of ovarian cancer in Indigenous/Others females. As one might expect, there were considerable numbers of secondary neoplasms of obscure origin. The usual tumours of childhood, e.g. retinoblastoma and nephroblastoma were observed. Not included in Table I are 3 adamantinomata, seen in a Chinese male, and in an Indigenous male and female. Six cancers arising in the conjunctiva of the eyelid are included with the skin neoplasms (ISC No. 191); these were found in males, 2 Chinese and 4 Indigenous/Others.

The age-specific minimum incidence rates, all sites, indicate in general a gradual rise with advancing age for all groups. The crude and age-specific rates for the Indigenous/Others group are considerably lower than those for Chinese.

### DISCUSSION

The results in Table I indicate clearly under-reporting, the relative inadequacy of the medical care facilities, and the excess of cancers of the more accessible parts of the body which one would expect in such circumstances. A high proportion of skin cancers was also observed in neighbouring Sarawak. It is of great interest to note that, as in Sarawak, nasopharyngeal cancer seems to be of the same order of frequency in the indigenous peoples as it is in Chinese (Muir and Oakley, 1967). Surprisingly no cases of nasopharyngeal cancer were seen in Chinese women. Although not statistically significant, this finding merits particular attention in subsequent investigations. It has already been noted that the Sabah Chinese live in the towns or in the countryside around the towns, whereas the other races live in the rural areas.

The chewing of betel is a common habit of the Kadazan and Bajau groups. Despite this, as in Sarawak, (Muir and Oakley, 1966) the relative frequency of oral cavity cancer is fairly low. The quid chewed (Muir and Kirk, 1960) includes the areca nut, the leaf of the betel vine and lime prepared from shells and coral. Orme (1914) gives an entertaining account of his first chew of " this abomination "— a Borneo betel quid. Further work is needed to establish whether tobacco is commonly added.

As one might expect from a young population, there is a considerable number of connective tissue neoplasms. The high frequency of malignant lymphoma in the Indigenous is striking: of the 18 in males, 9 were classified as lymphosarcoma, 7 as reticulum cell sarcoma and 2 as Hodgkin's disease; corresponding figures for females were 2, 3 and 3 respectively. A high frequency of such cancers was also seen in Sarawak Dayaks (Muir and Oakley, 1966).

Comparison of cancer relative frequency data is a perilous exercise. Such data may be biased by under-reporting and selection and by differences in the age structure of the universe whence the series were drawn. The age factor may be corrected by the use of an age-standardized cancer ratio or ASCAR (Tuyns, 1968). Such ratios are given in the table: they are based on rather small numbers. It should be noted that in this series a case in the group 65+ carries a heavy weight in the ASCAR.

The age-standardization results in certain changes in the relative frequency of several sites—nasopharyngeal cancers become slightly less prominent, skin cancers more so. Despite correction for age, malignant lymphoma in Indigenous/Others males is very prominent. The relative frequency of breast cancer in Chinese females and of ovarian cancers in Indigenous/Others females is high. These frequencies are considerably increased in the ASCAR due to the weight of the patients 65 years of age and over.

The crude minimum incidence rates for Chinese in Table I are approximately 2/3 of those in the Chinese population of Singapore (Doll, Payne and Waterhouse,

TABLE I.—*Relative Frequency of* 380 *Biopsied Cancers in Sabah Chinese and Indigenous/Others by Sex, Site and Broad Age-Group, together with the Age-Standardized Cancer Ratio (ASCAR), the* 1960 *Census Population and Age-Specific Minimum Incidence Rates per* 100,000 *per Annum*

| | | Chinese | | | | | | | | | | | | | |
|---|---|---|---|---|---|---|---|---|---|---|---|---|---|---|---|
| | | Male | | | | | | | Female | | | | | | |
| I.S.C. No. (7th Rev.)† | Site | 0–14 | 15–34 | 35–64 | 65+ | All ages | Rel. freq. % | ASCAR | 0–14 | 15–34 | 35–64 | 65+ | All ages | Rel. freq. % | ASCAR |
| 140 | Lip | — | — | 1 | — | 1 | 1·0 | 0·8 | — | — | — | — | — | — | — |
| 141 | Tongue | — | — | 2 | — | 2 | 2·0 | 1·5 | — | — | 1 | — | 1 | 1·4 | 0·9 |
| 142 | Salivary gland | — | 1 | — | — | 1 | 1·0 | 0·7 | — | 1 | 1 | — | 2 | 2·8 | 1·9 |
| 143, 4 | Mouth | — | — | 2 | 1 | 3 | 3·0 | 3·4 | — | — | 2 | — | 2 | 2·8 | 1·7 |
| 146 | Nasopharynx | — | — | 8 | 1 | 9 | 8·9 | 7·9 | — | — | — | — | — | — | — |
| 151 | Stomach | — | 2 | 10 | 1 | 13 | 12·9 | 10·8 | — | 1 | 1 | — | 2 | 2·8 | 1·9 |
| 153 | Colon | — | 1 | 1 | — | 2 | 2·0 | 1·5 | — | — | 3 | 1 | 4 | 5·6 | 9·3 |
| 154 | Rectum | — | 1 | 2 | 2 | 5 | 5·0 | 5·8 | — | — | 5 | — | 5 | 6·9 | 4·3 |
| 155·0 | Liver | — | — | 5 | 1 | 6 | 5·9 | 5·6 | — | — | — | — | — | — | — |
| 157 | Pancreas | — | — | 1 | — | 1 | 1·0 | 0·8 | — | — | — | — | — | — | — |
| 162, 3 | Lung | — | — | 1 | 1 | 2 | 2·0 | 2·6 | — | — | — | — | — | — | — |
| 170 | Breast | — | — | — | — | — | — | — | — | — | 8 | 2 | 10 | 13·9 | 20·3 |
| 171 | Cervix uteri | — | — | — | — | — | — | — | — | — | 10 | — | 10 | 13·9 | 8·7 |
| 172 | Corpus uteri | — | — | — | — | — | — | — | — | — | 8 | — | 8 | 11·1 | 6·9 |
| 173 | Choriocarcinoma | — | — | — | — | — | — | — | — | — | 1 | — | 1 | 1·4 | 0·9 |
| 175 | Ovary | — | — | — | — | — | — | — | 1 | 1 | 2 | — | 4 | 5·6 | 4·0 |
| 177 | Prostate | — | — | — | — | — | — | — | — | — | — | — | — | — | — |
| 178 | Testis | 1 | 1 | — | — | 2 | 2·0 | 1·7 | — | — | — | — | — | — | — |
| 179·0 | Penis | — | — | — | 2 | 2 | 2·0 | 3·6 | — | — | — | — | — | — | — |
| 180 | Kidney | 1 | 1 | — | — | 2 | 2·0 | 1·7 | 1 | — | — | — | 1 | 1·4 | 1·3 |
| 181·0 | Bladder | — | — | 2 | 3 | 5 | 5·0 | 7·0 | — | — | — | — | — | — | — |
| 190 | Melanoma skin | — | — | — | — | — | — | — | — | — | — | — | — | — | — |
| 191 | Other skin* | — | — | 9 | 7 | 16 | 15·8 | 19·6 | — | — | 3 | 2 | 5 | 6·9 | 15·9 |
| 192 | Eye | 2 | — | — | — | 2 | 2·0 | 2·0 | — | — | — | — | — | — | — |
| 194 | Thyroid | — | 2 | — | 1 | 3 | 3·0 | 3·2 | — | 3 | 2 | 1 | 6 | 8·3 | 11·4 |
| 196 | Bone | 1 | — | — | — | 1 | 1·0 | 1·0 | 1 | — | — | — | 1 | 1·4 | 1·3 |
| 197 | Conn. tissue | — | 1 | 1 | — | 2 | 2·0 | 1·5 | — | 3 | 2 | — | 5 | 6·9 | 4·7 |
| 200, 1 | Malig. lymphoma | — | 1 | 1 | 2 | 4 | 4·0 | 5·1 | 1 | — | 2 | — | 3 | 4·2 | 3·0 |
| 204 | Leukaemia | — | — | 1 | — | 1 | 1·0 | 0·8 | — | 1 | — | — | 1 | 1·4 | 1·0 |
| Sec. Ca; Carcinomatosis | | — | 4 | 12 | — | 16 | 15·8 | 11·8 | — | — | 1 | — | 1 | 1·4 | 0·9 |
| 140–205 | All sites | 5 | 15 | 59 | 22 | 101 | — | — | 4 | 10 | 52 | 6 | 72 | — | — |
| Population ('000) | | 25 | 15 | 14 | 2 | 56 | — | — | 24 | 14 | 10 | 2 | 50 | — | — |
| Rates per 100,000 p.a. | | 5 | 25 | 105 | 275 | 45 | — | — | 4 | 18 | 130 | 75 | 36 | — | — |

* See text. † WHO, 1957. ‡ Includes one larynx cancer. § Includes one brain cancer.

*Note:* There were no cases of oesophageal cancers.

| I.S.C. No. (7th Rev.)† | Site | Indigenous/Others — Male 0–14 | 15–34 | 35–64 | 65+ | All ages | Rel. freq. % | ASCAR | Female 0–14 | 15–34 | 35–64 | 65+ | All ages | Rel. freq. % | ASCAR |
|---|---|---|---|---|---|---|---|---|---|---|---|---|---|---|---|
| 140 | Lip | — | — | — | — | — | — | — | — | — | — | — | — | — | — |
| 141 | Tongue | — | — | 1 | — | 1 | 0·8 | 0·8 | — | — | — | 1 | 1 | 1·1 | 6·7 |
| 142 | Salivary gland | 1 | 1 | 1 | — | 3 | 2·5 | 1·7 | — | — | 1 | — | 1 | 1·1 | 1·1 |
| 143, 4 | Mouth | — | — | 1 | 1 | 2 | 1·7 | 3·4 | — | — | 2 | — | 2 | 2·3 | 2·1 |
| 146 | Nasopharynx | 1 | 4 | 6 | — | 11 | 9·2 | 6·2 | — | 2 | 1 | — | 3 | 3·4 | 1·8 |
| 151 | Stomach | — | — | 6 | 1 | 7 | 5·9 | 7·2 | — | — | 2 | — | 2 | 2·3 | 2·1 |
| 153 | Colon | — | 1 | — | 1 | 2 | 1·7 | 3·0 | — | 1 | — | — | 1 | 1·1 | 0·4 |
| 154 | Rectum | — | — | 1 | — | 1 | 0·8 | 0·8 | — | — | — | — | — | — | — |
| 155·0 | Liver | — | — | 4 | — | 4 | 3·4 | 3·0 | — | 1 | — | — | 1 | 1·1 | 0·4 |
| 157 | Pancreas | — | — | 1 | — | 1 | 0·8 | 0·8 | — | — | — | — | — | — | — |
| 162, 3 | Lung | — | — | 2‡ | — | 2 | 1·7 | 1·5 | — | — | — | — | — | — | — |
| 170 | Breast | — | — | 1 | 1 | 2 | 1·7 | 3·4 | — | 2 | 4 | — | 6 | 6·8 | 5·0 |
| 171 | Cervix uteri | — | — | — | — | — | — | — | 1 | 2 | 8 | 1 | 12 | 13·6 | 16·4 |
| 172 | Corpus uteri | — | — | — | — | — | — | — | — | 2 | — | 1 | 3 | 3·4 | 7·4 |
| 173 | Choriocarcinoma | — | — | — | — | — | — | — | — | 4 | — | — | 4 | 4·6 | 1·4 |
| 175 | Ovary | — | — | — | — | — | — | — | — | 2 | 7 | 2 | 11 | 12·5 | 21·5 |
| 177 | Prostate | — | — | — | 1 | 1 | 0·8 | 2·7 | — | — | — | — | — | — | — |
| 178 | Testis | — | 4 | — | — | 4 | 3·4 | 1·1 | — | — | — | — | — | — | — |
| 179·0 | Penis | — | — | 1 | — | 1 | 0·8 | 0·8 | — | — | — | — | — | — | — |
| 180 | Kidney | — | — | 1 | — | 1 | 0·8 | 0·8 | 2 | — | — | — | 2 | 2·3 | 0·8 |
| 181·0 | Bladder | — | — | 1 | 2 | 3 | 2·5 | 6·1 | — | — | — | 1 | 1 | 1·1 | 6·7 |
| 190 | Melanoma skin | — | 4 | 1 | — | 5 | 4·2 | 1·9 | — | — | — | — | — | — | — |
| 191 | Other skin* | — | 8 | 10 | 2 | 20 | 16·8 | 15·1 | — | 2 | 6 | — | 8 | 9·1 | 7·1 |
| 192 | Eye | 2§ | — | 1 | — | 3 | 2·5 | 2·0 | 3 | — | — | — | 3 | 3·4 | 1·3 |
| 194 | Thyroid | — | — | — | — | — | — | — | — | 1 | 5 | — | 6 | 6·8 | 5·7 |
| 196 | Bone | — | 3 | 1 | — | 4 | 3·4 | 1·6 | 1 | — | 1 | — | 2 | 2·3 | 1·5 |
| 197 | Conn. tissue | 2 | 2 | 4 | — | 8 | 6·7 | 4·8 | 2 | 3 | 1 | — | 6 | 6·8 | 3·0 |
| 200, 1 | Malig. lymphoma | 1 | 7 | 9 | 1 | 18 | 15·1 | 12·0 | 2 | 4 | 2 | — | 8 | 9·1 | 4·4 |
| 204 | Leukaemia | — | — | 1 | — | 1 | 0·8 | 0·8 | — | 1 | — | — | 1 | 1·1 | 0·4 |
| Sec. Ca; Carcinomatosis | | 1 | 2 | 6 | 5 | 14 | 11·8 | 19·0 | 1 | 1 | 2 | — | 4 | 4·6 | 2·9 |
| 140–205 | All sites | 8 | 36 | 60 | 15 | 119 | — | — | 12 | 28 | 42 | 6 | 88 | — | — |
| Population ('000) | | 76 | 61 | 40 | 3 | 180 | — | — | 72 | 57 | 35 | 3 | 168 | — | — |
| Rates per 100,000 p.a. | | 3 | 15 | 38 | 125 | 17 | — | — | 4 | 12 | 30 | 50 | 13 | — | — |

* See text. † WHO, 1957. ‡ Includes one larynx cancer. § Includes one brain cancer.

*Note:* There were no cases of oesophageal cancers.

1966) and are slightly higher than those in Sarawak Chinese (Muir and Oakley, 1966). The crude minimum rates for Indigenous/Others are somewhat lower than those for Sarawak Dayaks. At this juncture, it would appear preferable to regard the low crude rates and the difference in overall rates between the Chinese and Indigenous peoples as being due to under-reporting until proved otherwise.

It should be emphasized that the above comments are based on a very small number of cases and at best constitute informed speculation. From the aetiological point of view, it would appear worthwhile endeavouring to confirm the apparent racial distribution of nasopharynx cancer and the apparently high frequency of malignant lymphoma and ovarian cancer in Indigenous/Others males and females respectively. There was no histological similarity between these lymphoid and ovarian cancers.

Despite the manifest shortcomings of the present data from Sabah it is of considerable importance that the cancer patterns of this, and other similar multiracial groups, be defined and investigated before the present fairly sharp difference in the way of life and environment of the various segments of the population become too blurred.

### SUMMARY AND CONCLUSIONS

The geography of tropical Sabah, the way of life of the principal communities (Chinese, Indigenous and Others) who live there, and sources of bias in the collection of cancer statistics are outlined. Under-diagnosis is likely as hospitals are few and far apart, communications are poor, necropsies very rare and there is but one medical practitioner per 10,000 population.

In 1963–66 all biopsies taken in Sabah were sent to the Army Command Laboratory, Singapore. The records of all tissues submitted over a period of 4 years were checked and 380 cancers seen in Chinese and Indigenous/Others tabulated by sex, site, broad age-group and relative frequency.

Crude minimum incidence rates relating the number of biopsied cancers to the population-at-risk are very low; 45 and 36, and 17 and 13, per 100,000 per annum for Chinese and Indigenous/Others males and females respectively.

There is a preponderance of cancers from the more accessible sites and an excess of cancers for which the primary site was not established, findings suggesting a large measure of under-diagnosis.

Skin and nasopharynx cancers appear to be common in both Indigenous and Chinese. There was a considerable excess of malignant neoplasms of the lymphoid tissues in males of the Indigenous/Others group and of ovarian cancer in the Indigenous/Others females, differences which remain after correction for age to the age distribution of a large series of cancer cases from many parts of the world. There was no histological similarity between the ovarian and the lymphoid cancers. These patterns, although based on small numbers of cases, show certain similarities to those found in contiguous Sarawak (Muir and Oakley, 1966).

It is important that the differences observed be confirmed and investigated before the present fairly sharp differences in the way of life and environment of the various segments of the population become too blurred.

It is a pleasure to record the assistance of Brigadier H. C. Jeffrey (late R.A.M.C.) and Professor K. Shanmugaratnam, Singapore. Mr. B. C. Foo prepared Fig. 1.

Dr. D. M. Cameron, Director of Medical Services, Sabah, and the Ministry of Defence, kindly gave permission to publish.

### REFERENCES

ANON.—(1965) *Rep. med. Dep., Sabah, Malaysia*, for the Year 1963–1964. (Government Printing Office.)

DOLL, R., PAYNE, P. AND WATERHOUSE, J.—(1966) ‘Cancer Incidence in Five Continents’. (Publication of the International Union against Cancer.) Berlin-Heidelberg–New York (Springer-Verlag).

JONES, W. L.—(1962) North Borneo. Report on the Census of Population taken on 10th August 1960. Kuching, Sarawak (Government Printing Office).—(1966) ‘The Population of Borneo. A Study of the Peoples of Sarawak, Sabah and Brunei’. London (Athlone Press).

MUIR, C. S. AND KIRK, R.—(1960) *Br. J. Cancer*, **14**, 597.

MUIR, C. S. AND OAKLEY, W. F.—(1966) *Br. J. Cancer*, **20**, 217.—(1967) *J. Lar. Otol.*, **81**, 197.

ORME, W. B.—(1914) *Br. med. J.*, i, 325.

POLUNIN, I.—(1958) *Lancet*, ii, 1005.

TUYNS, A. T.—(1968) *Int. J. Cancer*, **3**, 397.

WORLD HEALTH ORGANIZATION—(1957) ‘Manual of the International Statistical Classification of Diseases, Injuries and Causes of Death’, 7th Revision, Vol. 1, Geneva (World Health Organization).